# Prospective Comparative Study of Functional Outcomes of Distal Extra-articular Tibia Fracture Fixed With Intramedullary Nailing Versus Locking Compression Plating

**DOI:** 10.7759/cureus.102995

**Published:** 2026-02-04

**Authors:** Faisal M Mulla, Chandrashekar M, Sneha Achar, Sattick Chatterjee, B G Sagar

**Affiliations:** 1 Orthopaedics, Adichunchanagiri Institute of Medical Sciences, B.G. Nagara, IND; 2 Department of Orthopaedics, Employees' State Insurance Corporation Medical College &amp; Post Graduate Institute of Medical Science and Research, Bangalore, IND

**Keywords:** aofas score, distal tibial fractures, extra-articular tibia fracture, functional outcomes, intramedullary nailing, locking compression plate

## Abstract

Introduction

Distal tibial fractures are prevalent long bone injuries that pose difficulties owing to insufficient soft tissue coverage and their closeness to the ankle joint. The selection of the fixing method continues to be contentious, especially for extra-articular fractures. This study sought to investigate extra-articular distal tibial fractures, compare intramedullary nailing (IMN) with minimally invasive plate osteosynthesis (MIPO), evaluate their clinical, radiological, and functional outcomes, and identify the problems linked to each procedure.

Methodology

This prospective comparative study was conducted from March 2022 to March 2025 and involved 40 patients with extra-articular distal tibial fractures. Patients were categorized into two cohorts: 20 received IMN and 20 underwent locking compression plating (LCP). The average patient age was 42.2 years in the IMN group and 49.1 years in the LCP group. Both genders were represented, and laterality was nearly equilibrated. Patients received assessments prior to, during, and following the operation. A clinical examination was conducted with the American Orthopaedic Foot & Ankle Society (AOFAS) score, and radiological evaluation was carried out through X-ray imaging. Functional outcome was assessed using the AOFAS ankle-hindfoot score (pain 40, function 50, alignment 10; total 100). Outcomes were categorized as excellent (>90), good (81-90), fair (71-80), and poor (<70).

Results

At the one-year follow-up, all fractures had healed. In the IMN group, seven patients (35%) attained outstanding outcomes, whereas 13 patients (65%) received good outcomes. Within the LCP cohort, four (20%) patients achieved great outcomes, 12 (60%) attained good outcomes, three (15%) experienced fair outcomes, and one (5%) exhibited a bad outcome. Complications arose in four patients in the LCP group (two superficial wound infections, one deep infection, and one persistent ankle soreness) and in two patients in the IMN group (both superficial wound infections). No malunion or nonunion was detected. Good outcome in this study refers to an AOFAS score between 81 and 90 at the 12-month follow-up.

Conclusion

IMN and LCP yielded advantageous long-term results for extra-articular distal tibial fractures. IMN exhibited benefits in reduced operational duration, diminished complications, and enhanced functional outcomes during the early follow-up phase, however long-term results were similar across groups. More extensive investigations with prolonged follow-up are required to corroborate these results.

## Introduction

Fractures in the distal tibial metadiaphyseal region are prevalent and usually occur due to high-energy trauma, including vehicular collisions, torsional injuries, and falls from elevation. They constitute roughly 10% of all tibial fractures [1,2]. Distal tibial metaphysis is defined by constructing a square, with the length of the sides defined by the widest portion of the tibial plafond. Non-articular fractures, which do not penetrate the joint surface, contrast with pilon fractures in terms of mechanism and prognosis [3,4]. These fractures provide specific problems for surgical intervention owing to their closeness to the ankle joint and insufficient soft tissue covering. They are often linked to displacement, comminution, and soft tissue damage [5]. Furthermore, distal tibial metaphyseal fractures categorized as Arbeitsgeminschaft fur Osteosynthesefragen (AO) type 43A1, 43A2, and 43A3 are particularly susceptible to delayed union and nonunion due to precious blood supply [4]. These issues require meticulous selection of fixing methods to enhance functional results. Hansmann initially documented the utilization of plates for treating tibial fractures in Germany during the 1880s. Karlström (1972) subsequently reported positive outcomes in 90% of 135 tibial fractures managed with plating [6]. Locking compression plating (LCP) offers angular stability, reduces periosteal stripping, and is especially beneficial for osteopenic bone and comminuted fractures. When utilized alongside minimally invasive plate osteosynthesis (MIPO), LCP safeguards soft tissue integrity and sustains alignment in distal tibial fractures. Nonetheless, the requirement for surgical exposure still entails risks of wound complications and infection, particularly in individuals with impaired soft tissue. Intramedullary nailing (IMN) is a load-sharing fixation method that promotes rapid healing via extensive callus production and enables early mobilization. Contemporary interlocking nails offer enhanced stability and diminish malalignment in comparison to previous models. Distal tibial fractures may present technical challenges for IMN due to problems in attaining precise alignment, and may lead to consequences such as malunion and anterior knee discomfort. While IMN and LCP are recognized treatments for distal extra-articular tibial fractures, the most effective approach remains contentious due to discrepancies in union rates, complication profiles, and functional recovery [7]. A notable deficiency in the literature is the scarcity of prospective studies that directly compare these two modalities, especially for long-term functional outcomes and complication rates. This study aimed to assess extra-articular distal tibial fractures, compare IMN with MIPO, analyze their clinical, radiological, and functional outcomes, and identify the complications linked to each technique.

## Materials and methods

Source of data

The study was conducted on patients with distal tibia fractures admitted to the Adichunchanagiri Institute of Medical Sciences, B.G. Nagara, Karnataka, India.

Study design

This study was a prospective research project that included consecutive cases of distal tibia fractures occurring between March 2023 and March 2025. Ethical approval was secured from the Institutional Ethics Committee of the Adichunchanagiri Institute of Medical Sciences (approval no. AIMS/IEC/108/2023) before the commencement of the project.

Inclusion and exclusion criteria

Patients over 18 years of age, regardless of sex, with closed distal extra-articular tibia fractures categorized according to the AO classification system 43A1, 43A2, and 43A3 were included. Patients with pathological, open, or segmental fractures; ignored old fractures; intra-articular extension; peripheral vascular disease; or diabetes mellitus with HbA1c levels exceeding seven were excluded. The distal tibia is delineated as the region within the two Müller squares of the ankle joint, where the proximal and distal segments of long bones are characterized by a square equivalent in length to the broadest part of the epiphysis.

Sample size and grouping

Consecutive sampling approach was adopted. Forty patients meeting the criteria were enrolled. Surgical intervention was conducted utilizing either minimally invasive plate osteosynthesis (MIPO) or intramedullary nailing (IMN), contingent upon the surgeon's discretion. Twenty patients received treatment using MIPO, while another twenty were treated with IMN. The collected data encompassed age, sex, side, mode of injury, complication rates, surgical duration, union time, and American Orthopaedic Foot & Ankle Society (AOFAS) scores. In the IMN group, a standard infrapatellar approach was used. Closed reduction was achieved under fluoroscopy. An intramedullary interlocking nail was inserted after guidewire placement and canal preparation as required. At least two distal interlocking screws were used to enhance distal fixation stability. Reduction and alignment were checked in both AP and lateral views to minimize varus/valgus and sagittal plane malalignment.

Pre-operative evaluation

Patients underwent clinical evaluation for the mechanism and speed of damage, state of soft tissues, classification of fractures, neurovascular integrity, and occurrence of compartment syndrome. Radiographs (anteroposterior and lateral views) were acquired, and fractures were categorized according to the AO classification system. Temporary immobilization was achieved using an above-knee slab, accompanied by appropriate analgesia, until permanent fixation could be performed after anesthetic clearance. 

Functional outcome was measured using the American Orthopaedic Foot & Ankle Society (AOFAS) ankle-hindfoot score [8] at three, six, and 12 months. The score includes pain (40 points), function (50 points), and alignment (10 points), with a total of 100 points. Functional outcome categories were defined as excellent (>90), good (81-90), fair (71-80), and poor (<70).

Radiological outcomes were assessed using standard anteroposterior and lateral radiographs obtained at each follow-up visit. Union was defined as bridging callus across at least three of four cortices on anteroposterior (AP) and lateral views, along with absence of pain/tenderness at the fracture site. Malalignment was defined as varus/valgus >5° and/or procurvatum/recurvatum >10°.

Pre-operative investigations

Standard assessments comprised complete blood count, urinalysis, blood grouping and Rh typing, coagulation profile, renal and hepatic function tests, serum electrolytes, random blood glucose, and viral markers (Human Immunodeficiency Virus (HIV) types I and II, Hepatitis B surface antigen (HBsAg), and Hepatitis C virus (HCV)) with consent. Imaging encompassed leg radiographs, electrocardiograms, chest X-rays (posteroanterior (PA) view), and echocardiography if warranted.

Intraoperative procedure

**Figure 1:**
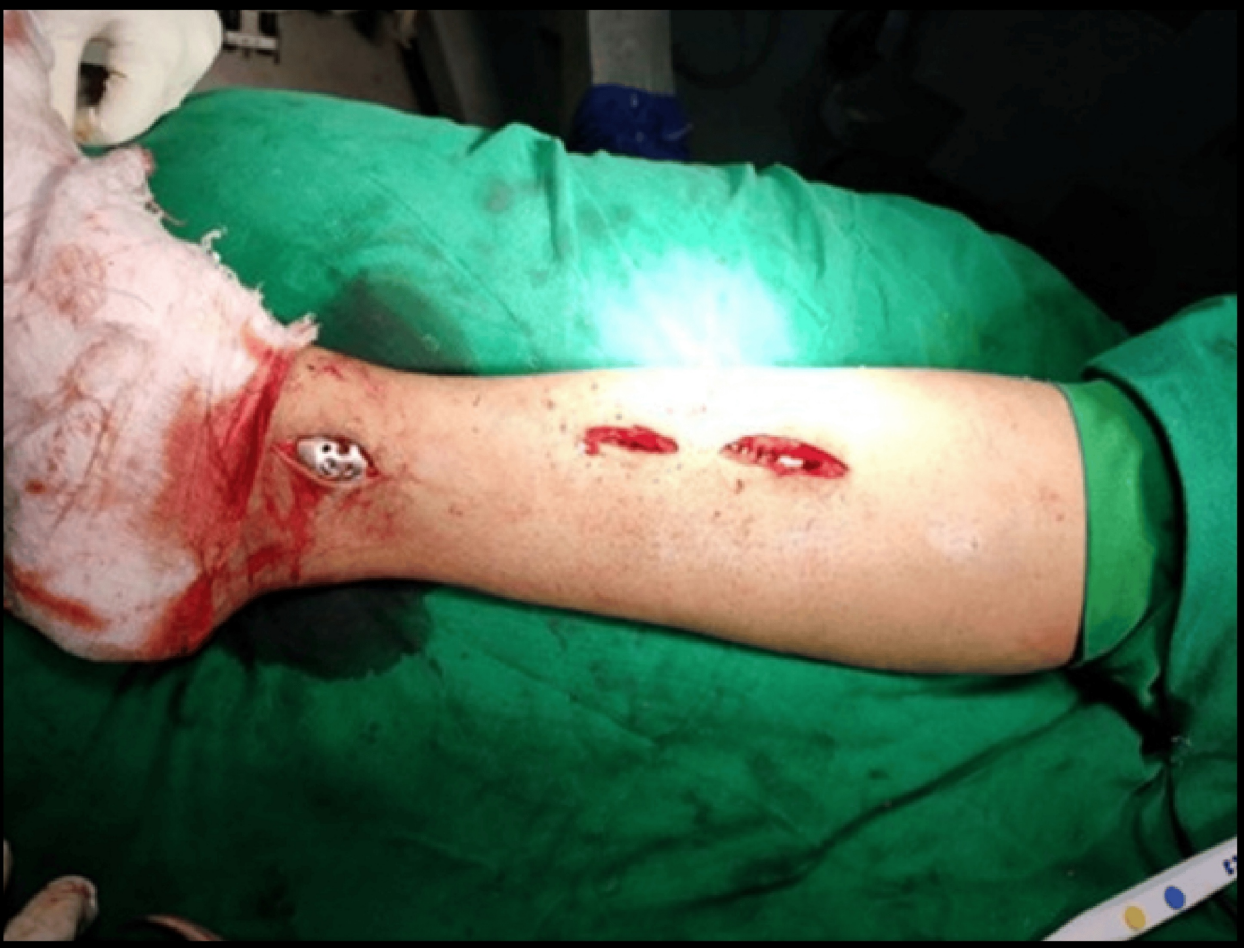
Final fixation of plate with screws

**Figure 2:**
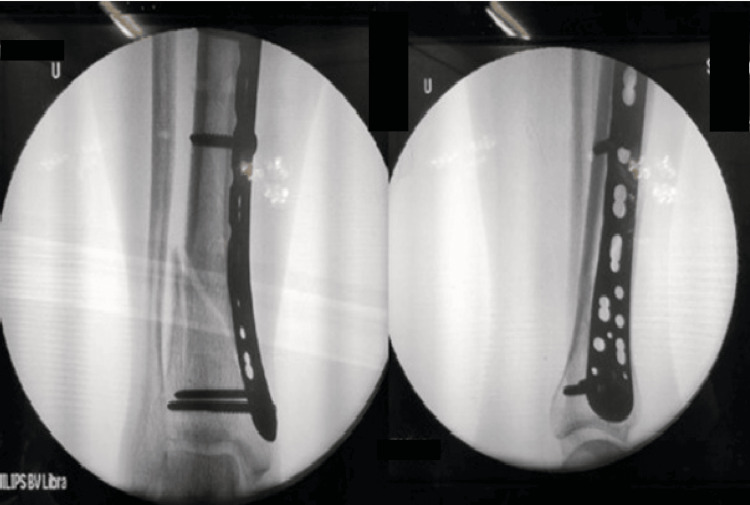
Fluoroscopic images of minimally invasive plate osteosynthesis (MIPO)

**Figure 3:**
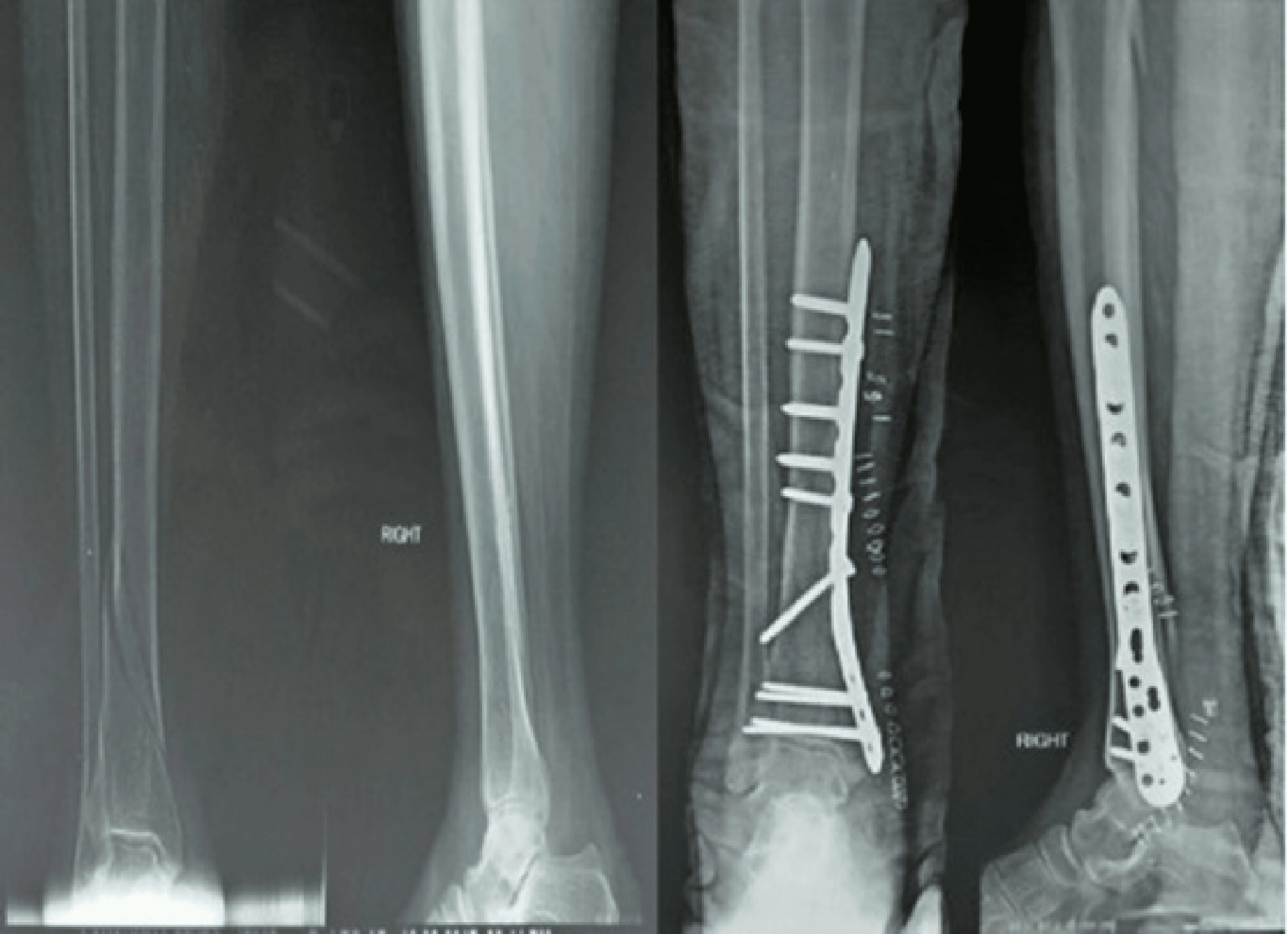
Pre- and postoperative images of minimally invasive plate osteosynthesis (MIPO)

Patients were positioned supine under spinal anesthesia. In the MIPO group, reduction was accomplished using a typical medial approach and fixation with a locking compression plate under C-arm supervision. In the IMN group, closed reduction and intramedullary interlocking nailing were executed. The operative period was defined as the interval from skin incision to the completion of skin closure. Fibula fractures occurring within 7 cm of the lateral malleolus tip were stabilized with plating prior to tibial fixation (Figures 1-3).

Postoperative care

All patients received standard postoperative antibiotics and analgesia as per institutional protocol. Early ankle and knee range-of-motion exercises were initiated as tolerated.

IMN Group

Patients were encouraged for early mobilization. Non-weight-bearing ambulation with walker/crutches was started from postoperative day one or two, depending on pain and stability. Partial weight bearing was initiated once early radiological callus was seen and pain was minimal, and progressed to full weight bearing based on clinical and radiological evidence of union.

MIPO/LCP Group

Limb elevation and soft-tissue care were emphasized. Ankle range-of-motion exercises were initiated early. Non-weight-bearing ambulation was started after the wound condition permitted. Weight-bearing progression followed radiological callus formation and the surgeon's assessment. Suture removal was done on postoperative day 14. Follow-up was scheduled at three, six, and 12 months.

Follow-up and functional assessment

Patients were monitored at three, six, and 12 months. The clinical assessment encompassed pain and soft tissue evaluation, whereas the radiological assessment involved fracture alignment, reduction, and union. The union was characterized as the amalgamation of three of the four cortices on AP and lateral radiographs. Healing that occurs within six to nine months is classified as delayed union, while a lack of healing after nine months is classified as nonunion. The AOFAS score was utilized to assess functional results, encompassing pain (40 points), function (50 points), and alignment (10 points), with a total possible score of 100 points. Scores were classified as exceptional (>90), decent (81-90), fair (71-80), or poor (<70).

Statistical analysis

Data analysis was conducted via IBM SPSS Statistics for Windows, Version 25 (Released 2017; IBM Corp., Armonk, New York, United States). Continuous variables (age, operative duration, and union time) were presented as mean ± SD and analyzed using independent t-tests. Categorical factors (gender, injury side, fracture type, fibula involvement, and sequelae) were examined with the chi-square test. AOFAS scores were analyzed at three time intervals (three, six, and 12 months) utilizing repeated-measures analysis of variance (ANOVA), with p<0.05 being statistically significant. The interval between injury and definitive surgery (trauma-to-surgery interval) was recorded for all patients. Surgery was performed once soft-tissue condition was satisfactory (wrinkle sign present and swelling controlled). The trauma-to-surgery interval was compared between groups and reported as mean ± SD.

## Results

A total of 40 patients were included, with 20 in the IMN group and 20 in the MIPO/LCP group. The mean age was 42.2 ± 11.9 years in the IMN group and 49.1 ± 11.2 years in the MIPO group. Gender distribution was comparable: the IMN group had 14 (70%) male patients and six (30%) female patients, while the MIPO group had 12 (60%) male patients and eight (40%) female patients. The side of injury was evenly distributed across both groups, with the former group showing nine (45%) left-sided and 11 (55%) right-sided cases, and the latter group showing nine (45%) left-sided and 11 (55%) right-sided cases. Fracture types differed significantly between groups (p<0.001): AO 43A1 was more common in the IMN group (n=13; 65%) compared to the MIPO group (n=1; 5%); AO 43A2 was more frequent in the MIPO group (n=10; 50%) compared to the IMN group (n=7; 35%); and AO 43A3 occurred only in former group (n=9; 45%). Fibula involvement was significantly higher in the MIPO group (n=15; 75%) compared to the IMN groups (n=3; 15%) (p<0.001). Most injuries resulted from road traffic accidents, with IMN accounting for 19 (95%) and MIPO for 17 (85%) cases (Table 1).

**Table 1:** Baseline characteristics of patients IMN: intramedullary nailing; MIPO: minimally invasive plate osteosynthesis; LCP: locking compression plating; χ²: chi-square statistic; t: independent t-test; *Indicates statistical significance (p<0.05).

Variables	IMN (N=20)	MIPO/LCP (N=20)	Total (N=40)	Test statistic	p-value
Age (years, mean ± SD)	42.2 ± 11.9	49.1 ± 11.2	45.7 ± 11.9	t = 0.52	0.604
Gender				χ² = 0.44	0.50
Male	14 (70%)	12 (60%)	26 (65%)		
Female	6 (30%)	8 (40%)	14 (35%)		
Side of injury				χ² = 0.00	1.00
Left	9 (45%)	9 (45%)	18 (45%)		
Right	11 (55%)	11 (55%)	22 (55%)		
Fracture type				χ² = 19.81	<0.001
43 A1	13 (65%)	1 (5%)	14 (35%)		
43 A2	7 (35%)	10 (50%)	17 (42.5%)		
43 A3	0 (0%)	9 (45%)	9 (22.5%)		
Fibula involvement				χ² = 14.54	<0.001*
Yes	3 (15%)	15 (75%)	18 (45%)		
No	17 (85%)	5 (25%)	22 (55%)		
History of the fall				χ² = 1.11	0.29
Road traffic accident	19 (95%)	17 (85%)	36 (90%)		
Self fall	1 (5%)	3 (15%)	4 (10%)		

The mean operative time was significantly shorter in the IMN group (69.0 ± 9.1 min) compared to the MIPO group (96.0 ± 17.9 min) (p<0.001). The mean time to union was similar between the groups (IMN: 15.6 ± 2.8 weeks; MIPO: 15.9 ± 2.9 weeks; p=0.701). All fractures united in both groups (100%). Postoperative complications were observed in two (10%) patients in the IMN group and four (20%) patients in the MIPO group (Table 2).

**Table 2:** Operative details and fracture union IMN: intramedullary nailing; MIPO: minimally invasive plate osteosynthesis; LCP: locking compression plating; χ²: chi-square statistic; t: independent t-test; *Indicates statistical significance (p<0.05).

Variables	IMN (N=20)	MIPO/LCP (N =20)	Mean difference	Test Statistic	p-value
Operative time (min, mean ± SD)	69.0 ± 9.1	96.0 ± 17.9	27	t = 6.10	<0.001*
Time to union (weeks, mean ± SD)	15.6 ± 2.8	15.9 ± 2.9	-0.35	t = 0.39	0.701
Rate of union	20 (100%)	20 (100%)	-		-
Complications				χ² = 0.37	0.54
Yes	2 (10%)	4 (20%)			
No	18 (90%)	16 (80%)			

Mean AOFAS scores were consistently higher in the IMN group at all follow-up points. At three months, scores were 77.35 ± 4.67 for the IMN group and 75.35 ± 5.39 for the MIPO group (p=0.218). At six months, the IMN group showed significantly higher scores (82.50 ± 4.85 vs. 79.05 ± 5.04; p=0.034), and at 12 months, the IMN group continued to outperform MIPO (88.50 ± 4.59 vs. 83.70 ± 7.75; p=0.024) (Table 3).

**Table 3:** Functional outcomes (AOFAS score) IMN: intramedullary nailing; MIPO: minimally invasive plate osteosynthesis; LCP: locking compression plating; ANOVA: Analysis of Variance; F-value: F-statistic measuring between group vs within group variance; df: degrees of freedom; AOFAS: American Orthopaedic Foot & Ankle Society; Repeated-measures ANOVA test used; *statistically significant (p<0.05).

Time	IMN (N=20)	MIPO/LCP (N =20)	Mean difference	F-value (df = 1,38)	p-value
3 months	77.35 ± 4.67	75.35 ± 5.39	2	F = 1.57	0.218
6 months	82.50 ± 4.85	79.05 ± 5.04	3.45	F = 4.79	0.034
12 months	88.50 ± 4.59	83.70 ± 7.75	4.80	F = 5.51	0.024

In the IMN group, seven (35%) patients had excellent and 13 (65%) had good outcomes. In the MIPO group, four (20%) were excellent, 12 (60%) good, three (15%) fair, and one (5%) poor. Overall, excellent outcomes were observed in 11 (27.5%), good in 25 (62.5%), fair in three (7.5%), and poor in one (2.5%) (Table 4).

**Table 4:** Functional outcomes (AOFAS score categories at 12 months) IMN: intramedullary nailing; MIPO: minimally invasive plate osteosynthesis; LCP: locking compression plating; AOFAS: American Orthopaedic Foot & Ankle Society.

Outcome scores	IMN (N=20)	MIPO/LCP (N =20)	Total (N=40)
Excellent (>90)	7 (35%)	4 (20%)	11 (27.5%)
Good (81 to 90)	13 (65%)	12 (60%)	25 (62.5%)
Fair (71 to 80)	0 (0%)	3 (15%)	3 (7.5%)
Poor (<70)	0 (0%)	1 (5%)	1 (2.5%)

Pain severity differed significantly between groups (p=0.031). In the IMN group, 18 (90%) patients reported mild pain and two (10%) moderate pain. In the MIPO group, four (20%) had no pain, 11 (55%) mild, and five (25%) moderate. Overall, four (10%) patients had no pain, 29 (72.5%) mild, and seven (17.5%) moderate (Table 5).

**Table 5:** Pain outcomes IMN: intramedullary nailing; MIPO: minimally invasive plate osteosynthesis; LCP: locking compression plating.

Pain severity	IMN (N=20)	MIPO/LCP (N =20)	Total (N=40)	Chi-square value	p-value
None	0 (0%)	4 (20%)	4 (10%)	6.97	0.031
Mild	18 (90%)	11 (55%)	29 (72.5%)		
Moderate	2 (10%)	5 (25%)	7 (17.5%)		

## Discussion

Distal extra-articular tibial fractures present with considerable management challenges owing to their proximity to the ankle joint, limited soft-tissue coverage, and elevated risk of complications. Numerous fixation techniques have been described, with IMN and LCP being the most commonly used. Treatment selection often depends on fracture morphology, surgeon preference, and patient factors. This study examined the functional and radiological outcomes of these two methods to determine their relative effectiveness.

Our findings indicated that IMN produced more excellent and good outcomes, while the plating group included fewer fair and poor results. These outcomes are consistent with Prakashappa et al., who reported improved functional outcomes in the nailing group (35% excellent, 65% good) compared with the plating group (20% excellent, 60% good, 15% fair, 5% poor) [9]. We observed superficial wound infections in both groups, with a higher incidence in the plating group, which aligns with their report of deep infection and chronic ankle pain. Neither study reported malunion or nonunion, indicating that both fixation methods achieve reliable union, while IMN may offer better functional outcomes with fewer wound-related complications.

The present study further confirmed that IMN resulted in a higher proportion of excellent and good outcomes than plating, with fewer wound-related morbidities and no cases of malunion or nonunion. These findings align with recent evidence from advanced fixation strategies. A 2024 study comparing bone transport using external fixators versus IMN for tibial bone defects reported improved functional outcomes, shorter union time, lower complication rates, and reduced external fixation duration with intramedullary fixation [10]. Although the clinical contexts differed, both studies emphasized the advantage of intramedullary fixation in achieving stable union with fewer sequelae than some alternative techniques.

Kadier et al. (2024) [11] evaluated 22 patients with tibial defects following fracture-related infection treated using LCP as sequential external fixation; they reported 100% union with predominantly excellent and good bone and functional outcomes despite prior surgeries and compromised soft tissues. Although their cohort comprised complex post-infective defects and ours comprised acute extra-articular fractures, both studies highlight the importance of stable internal fixation for bone union and rehabilitation. IMN may be preferable in uninfected cases without significant scarring, whereas sequential LCP may be more suitable for addressing significant bone defects and poor soft-tissue conditions.

Nath et al. (2023) [12] reported a shorter mean union time in the IMN group (18.45 ± 2.45 weeks) than in the MIPO group (20 ± 3.21 weeks) and slightly higher mean AOFAS scores in the IMN than the MIPO group (92.6 ± 5.41 vs. 91.2 ± 6.81). Both studies indicate that overall radiological and functional outcomes are comparable between IMN and LCP. Wound complications are more frequent with LCP/MIPO, supporting IMN’s advantage in reducing soft-tissue morbidity.

Daolagupu et al. (2017) [13] reported that IMN facilitated faster union, earlier weight bearing, and fewer complications than LCP. Our study also demonstrated superior early functional recovery and fewer wound-related complications with IMN, although both groups achieved acceptable union by one year.

Guo et al. [14] concluded that closed IMN and percutaneous plating are safe for distal tibial fractures, but IMN offers shorter operative and radiation times and easier implant removal. Our results echo these findings: IMN was associated with faster union, earlier weight bearing, and fewer wound complications.

Meta-analyses by Ekman et al. (2021) [15] and Li et al. (2024) [16] reported that IMN reduced operative time, expedited weight bearing, decreased union time, and lowered rates of deep infection, wound complications, and secondary procedures compared with LCP. Li et al. also noted higher rates of malunion and anterior knee pain after IMN; our cohort did not observe these complications, suggesting that meticulous surgical technique can mitigate such risks.

Guo et al. (2018) [17], Hu et al. (2019) [18], Attia et al. (2022) [19], and Asloum et al. (2014) [20] similarly documented fewer wound complications and comparable union rates with IMN versus plating, with variable reports regarding malalignment and knee pain. Puga et al. (2025) [21] showed that fibular IMN (fIMN) yields comparable operative time, functional scores, union rates, and reoperation rates to plating but with significantly fewer wound complications; our findings are concordant. Lin et al. (2024) [22] also reported fewer wound-related and non-wound-related complications with IMN. However, they observed suboptimal mortise reduction in some IMN cases, a finding not replicated in our series. Such heterogeneity likely reflects differences in fracture patterns, surgical technique, and patient selection.

Overall, the available evidence, including the present study, indicates that while IMN and plating achieve reliable union in extra-articular distal tibial fractures, IMN is associated with superior early functional recovery and reduced wound morbidity in many series. Further research is required to define which fracture patterns or clinical scenarios carry a higher risk of malalignment with IMN and to clarify when plating or combined approaches may be preferable.

Limitations

This study is limited by its small sample size, consecutive sampling rather than a formal power-based sample size calculation was used, which may restrict the ability to detect small but clinically relevant differences between treatment groups, relatively short follow-up of one year, unequal distribution of fracture subtypes between groups, and exclusive reliance on the AOFAS grading system without incorporating broader quality-of-life assessments. Additionally, coexisting knee osteoarthritis in some older patients may have influenced the functional scores. Large, multicenter trials with extended follow-up and multiple outcome measures are needed to more definitively establish the most effective fixation method for distal extra-articular tibial fractures.

## Conclusions

This study concludes that both IMN and MIPO using LCP are effective and reliable surgical options for the management of distal extra-articular tibial fractures, demonstrating high rates of fracture union and satisfactory long-term functional outcomes. Although both modalities achieved comparable radiological healing at one year, IMN offered several distinct advantages, including significantly shorter operative duration, reduced wound-related complications, decreased postoperative pain, and superior early functional recovery, as reflected by the higher AOFAS scores at the six- and 12-month follow-ups.

The results of this study reinforce the growing body of evidence that IMN is particularly beneficial in cases with favorable soft-tissue conditions and simple extra-articular fracture patterns. Conversely, MIPO remains a valuable alternative in fractures requiring direct or indirect reduction, in cases with metaphyseal comminution, or when intramedullary alignment may be technically challenging. Thus, the choice of fixation should be individualized, considering fracture morphology, soft-tissue status, the surgeon's expertise, and patient-specific factors.
